# 4-Chloro-2-[(*E*)-({4-[*N*-(3,4-dimethyl­isoxazol-5-yl)sulfamo­yl]phen­yl}iminio)meth­yl]phenolate

**DOI:** 10.1107/S1600536808005321

**Published:** 2008-02-27

**Authors:** Hazoor A. Shad, Zahid H. Chohan, M. Nawaz Tahir, Islam Ullah Khan

**Affiliations:** a Department of Chemistry, Bahauddin Zakariya University, Multan 60800, Pakistan; b University of Sargodha, Department of Physics, Sargodha, Pakistan; c Government College University, Department of Chemistry, Lahore, Pakistan

## Abstract

The title compound, C_18_H_16_ClN_3_O_4_S, is a Schiff base ligand in which the H atom of the hydr­oxy group has moved to the N atom of the imine group, resulting in a zwitterion. The structure is stabilized by an intra­molecular (N—H⋯O) and five inter­molecular (C—H⋯O, C—H⋯N and N—H⋯O) hydrogen bonds. The mol­ecules are linked to each other by hydrogen bonds and form a three-dimensional polymeric network. In addition, the aromatic rings are also involved in π–π inter­actions [centroid–centroid distance between aromatic rings = 3.7525 (11) Å].

## Related literature

For related literature, see: Chatterjee *et al.* (1982 ▶); Chohan *et al.* (2008 ▶); Hämäläinen *et al.* (1986 ▶); Nishimori *et al.* (2005 ▶).
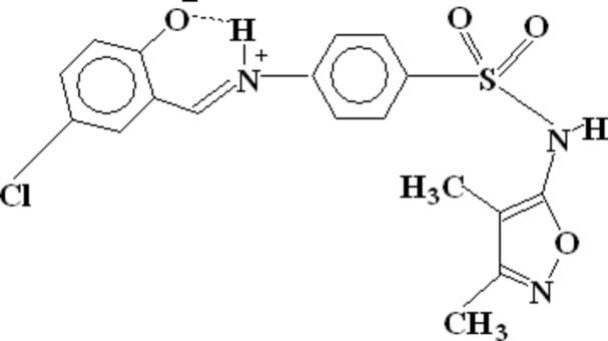


## Experimental

### 

#### Crystal data

C_18_H_16_ClN_3_O_4_S
*M* *_r_* = 405.85
Monoclinic, 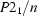

*a* = 15.1871 (6) Å
*b* = 7.2555 (3) Å
*c* = 16.6267 (7) Å
β = 94.081 (2)°
*V* = 1827.45 (13) Å^3^
*Z* = 4
Mo *K*α radiation
μ = 0.35 mm^−1^
*T* = 296 (2) K
0.30 × 0.25 × 0.20 mm

#### Data collection

Bruker Kappa APEXII CCD diffractometer
Absorption correction: multi-scan (*SADABS*; Bruker, 2005 ▶) *T*_min_ = 0.886, *T*_max_ = 0.935
18429 measured reflections
4669 independent reflections
3443 reflections with *I* > 2σ(*I*)
*R*_int_ = 0.028

#### Refinement

*R*[*F* ^2^ > 2σ(*F* ^2^)] = 0.042
*wR*(*F* ^2^) = 0.127
*S* = 1.04
3443 reflections
250 parameters
H atoms treated by a mixture of independent and constrained refinement
Δρ_max_ = 0.38 e Å^−3^
Δρ_min_ = −0.23 e Å^−3^

### 

Data collection: *APEX2* (Bruker, 2007 ▶); cell refinement: *APEX2*; data reduction: *SAINT* (Bruker, 2007 ▶); program(s) used to solve structure: *SHELXS97* (Sheldrick, 2008 ▶); program(s) used to refine structure: *SHELXL97* (Sheldrick, 2008 ▶); molecular graphics: *ORTEP-3 for Windows* (Farrugia, 1997 ▶) and *PLATON* (Spek, 2003 ▶); software used to prepare material for publication: *WinGX* (Farrugia, 1999 ▶) and *PLATON*.

## Supplementary Material

Crystal structure: contains datablocks global, I. DOI: 10.1107/S1600536808005321/pv2068sup1.cif

Structure factors: contains datablocks I. DOI: 10.1107/S1600536808005321/pv2068Isup2.hkl

Additional supplementary materials:  crystallographic information; 3D view; checkCIF report

## Figures and Tables

**Table 1:** Hydrogen-bond geometry (Å, °)

*D*—H⋯*A*	*D*—H	H⋯*A*	*D*⋯*A*	*D*—H⋯*A*
N1—H1⋯O1	0.87 (2)	1.85 (2)	2.574 (2)	140 (2)
C7—H7⋯N3^i^	0.93	2.54	3.429 (3)	161
C12—H12⋯O2^i^	0.93	2.59	3.391 (3)	145
C17—H17*C*⋯O3^ii^	0.96	2.57	3.516 (3)	168
C17—H17*B*⋯O1^iii^	0.96	2.38	3.276 (3)	156
N2—H2⋯O1^iii^	0.79 (2)	2.06 (2)	2.846 (2)	173 (2)

Symmetry codes: (i) 
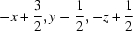
; (ii) 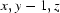; (iii) 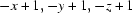.

**Fig. 1.**
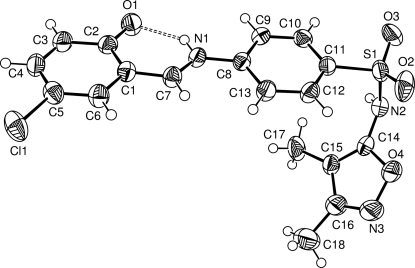
ORTEP-3 (Farrugia, 1997) drawing of (I) with the atom numbering scheme. The thermal ellipsoids are drawn at the 50% probability level. The intramolecular H-bonding is shown by dashed lines.

**Fig. 2.**
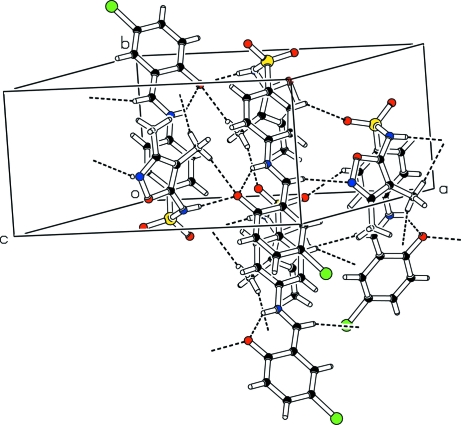
The unit cell packing of (I) (Spek, 2003) showing the interamolecular and intermolecular H-bonds leading to three-dimensional network.

## supplementary crystallographic information

### Comment

Sulfonamides have been found to possess a wide range of medicinal properties such as antibacterial, antitumor, diuretic, anti-carbonic anhydrase, hypoglycaemic, anti-thyroid and protease inhibitor (Nishimori *et al.*, 2005). In view of the versatile chemistry of various derivatives of sulfonamides, a continuous effort of synthesizing Schiff base ligands of substituted halogen salicylaldehyde and various sulfonamides (Chohan *et al.*, 2008) is in progress. In the same context, we herein report the structure of the title compound, (I), derived from the reaction of sulfisoxazole [*N*-(3,4-dimethyl-5-isoxazol)sulfanilamide] and 5-chlorosalicylaldehyde. The crystal structures of sulfisoxazole (Chatterjee *et al.*, 1982) and a bromo analog of (I) (Hämäläinen *et al.*, 1986) have already been published; the later is isomorphous with (I).

In the solid state the H-atom bonded to hydroxy group in (I) has shifted to N-atom of the Schiff base moiety, resulting in the formation of a zwitterion (Fig. 1). The bond distance C2—O1 [1.302 (2) Å] in (I) compares well with 1.295 (10) Å reported for the corresponding distance in the bromo-analog. The bond lengths in the aromatic rings in (I) are similar to the corresonding bond lengths reported in its bromo-isomorph. The range of bond angles around S-atom [104.86 (10)°-121.02 (11)°] in (I) is also the same as reported [104.5 (4)°-121.2 (4)°] in the bromo-analog (Hämäläinen *et al.*, 1986). The structure is stabilized by an intramolecular and five intermolecular H-bondings, the details of H-bonds are given in Table 1. The molecules linked to each other by H-bonds form a three-dimensional polymeric network (Fig. 2). In addition, π-π interactions between aromatic rings C8—C13 (ring A) and C1—C6 (ring B) are also observed with distance between the centers of gravity for the two rings CgA···CgB^iv^ (iv = *x*, *y* - 1, *z*) being Å. There also exists a π interaction between five-membered heterocyclic ring (ring C) and Cl1 with Cl1···CgC^v^ [v = *x* - 1/2, -*y* - 1/2, *z* - 1/2] distance of 3.5371 (11) Å. The dihedral angles between the rings A/B, A/C, B/C have values of 5.84 (9), 43.37 (11), and 43.29 (11)°, respectively.

### Experimental

An ethanol solution (15 ml) of sulfisoxazole (0.5346 g, 2 mmol) was added to a solution of 5-chlorosalicylaldehyde (0.3131 g, 2 mmol) in ethanol (10 ml). The reaction mixture was refluxed for 3 h. The solution was cooled to room temperature, filtered and volume reduced to about one-third using rotary evaporator. It was then allowed to stand for 13 days, after which orange-red crystals were obtained (m.p. 509 K).

### Refinement

The coordinates of H-atoms attached to N-atoms were refined. The rest of the H-atoms were positioned geometrically, with C—H = 0.93 and 0.96 Å for aromatic and methyl H-atoms, respectively, and constrained to ride on their parent atoms with *U*_iso_(H) = *xU*_eq_(C, N), where *x* = 1.5 for methyl H, and *x* = 1.2 for all other H atoms.

### Crystal data

**Table:**

C_18_H_16_ClN_3_O_4_S	*F*_000_ = 840
*M**_r_* = 405.85	*D*_x_ = 1.475 Mg m^−^^3^
Monoclinic, *P*2_1_/*n*	Melting point: 509 K
Hall symbol: -P 2yn	Mo *K*α radiation λ = 0.71073 Å
*a* = 15.1871 (6) Å	Cell parameters from 2465 reflections
*b* = 7.2555 (3) Å	θ = 2.2–28.7º
*c* = 16.6267 (7) Å	µ = 0.35 mm^−^^1^
β = 94.081 (2)º	*T* = 296 (2) K
*V* = 1827.45 (13) Å^3^	Prismatic, red
*Z* = 4	0.30 × 0.25 × 0.20 mm

### Data collection

**Table:**

Bruker Kappa APEXII CCD diffractometer	4669 independent reflections
Radiation source: fine-focus sealed tube	3443 reflections with *I* > 2σ(*I*)
Monochromator: graphite	*R*_int_ = 0.028
Detector resolution: 7.40 pixels mm^-1^	θ_max_ = 28.6º
*T* = 296(2) K	θ_min_ = 1.8º
ω scans	*h* = −20→20
Absorption correction: multi-scan(SADABS; Bruker, 2005)	*k* = −9→9
*T*_min_ = 0.886, *T*_max_ = 0.935	*l* = −22→22
18429 measured reflections	

### Refinement

**Table:**

Refinement on *F*^2^	Secondary atom site location: difference Fourier map
Least-squares matrix: full	Hydrogen site location: inferred from neighbouring sites
*R*[*F*^2^ > 2σ(*F*^2^)] = 0.042	H atoms treated by a mixture of independent and constrained refinement
*wR*(*F*^2^) = 0.127	*w* = 1/[σ^2^(*F*_o_^2^) + (0.0619*P*)^2^ + 0.5575*P*] where *P* = (*F*_o_^2^ + 2*F*_c_^2^)/3
*S* = 1.04	(Δ/σ)_max_ = 0.001
3443 reflections	Δρ_max_ = 0.38 e Å^−^^3^
250 parameters	Δρ_min_ = −0.22 e Å^−^^3^
Primary atom site location: structure-invariant direct methods	Extinction correction: none

### Special details

**Table:**

Geometry. All e.s.d.'s (except the e.s.d. in the dihedral angle between two l.s. planes) are estimated using the full covariance matrix. The cell e.s.d.'s are taken into account individually in the estimation of e.s.d.'s in distances, angles and torsion angles; correlations between e.s.d.'s in cell parameters are only used when they are defined by crystal symmetry. An approximate (isotropic) treatment of cell e.s.d.'s is used for estimating e.s.d.'s involving l.s. planes.
Refinement. Refinement of *F*^2^ against ALL reflections. The weighted *R*-factor *wR* and goodness of fit *S* are based on *F*^2^, conventional *R*-factors *R* are based on *F*, with *F* set to zero for negative *F*^2^. The threshold expression of *F*^2^ > σ(*F*^2^) is used only for calculating *R*-factors(gt) *etc*. and is not relevant to the choice of reflections for refinement. *R*-factors based on *F*^2^ are statistically about twice as large as those based on *F*, and *R*- factors based on ALL data will be even larger.

### Fractional atomic coordinates and isotropic or equivalent isotropic displacement parameters (Å^2^)

**Table:**

	*x*	*y*	*z*	*U*_iso_*/*U*_eq_	
Cl1	0.87797 (4)	−0.43370 (8)	0.58505 (4)	0.06440 (19)	
S1	0.60762 (4)	0.99136 (7)	0.32843 (3)	0.04563 (15)	
O1	0.62180 (10)	0.1417 (2)	0.64974 (8)	0.0504 (4)	
O2	0.68281 (11)	1.0347 (2)	0.28554 (11)	0.0678 (5)	
O3	0.56667 (13)	1.1279 (2)	0.37529 (10)	0.0676 (5)	
O4	0.59283 (10)	0.8115 (2)	0.14720 (8)	0.0529 (4)	
N1	0.67536 (10)	0.3239 (2)	0.52967 (9)	0.0368 (3)	
H1	0.6445 (13)	0.312 (3)	0.5715 (13)	0.044*	
N2	0.52879 (11)	0.9215 (2)	0.26153 (10)	0.0400 (4)	
H2	0.4845 (15)	0.908 (3)	0.2832 (13)	0.048*	
N3	0.59687 (15)	0.6461 (3)	0.10209 (12)	0.0666 (6)	
C1	0.73875 (12)	0.0376 (3)	0.57297 (11)	0.0378 (4)	
C2	0.68045 (13)	0.0167 (3)	0.63625 (11)	0.0395 (4)	
C3	0.68941 (15)	−0.1451 (3)	0.68403 (12)	0.0471 (5)	
H3	0.6540	−0.1608	0.7269	0.057*	
C4	0.74946 (15)	−0.2786 (3)	0.66788 (12)	0.0496 (5)	
H4	0.7533	−0.3850	0.6991	0.059*	
C5	0.80496 (13)	−0.2571 (3)	0.60509 (12)	0.0449 (4)	
C6	0.80079 (12)	−0.1021 (3)	0.55890 (12)	0.0438 (4)	
H6	0.8389	−0.0879	0.5179	0.053*	
C7	0.73313 (12)	0.1937 (3)	0.52144 (11)	0.0392 (4)	
H7	0.7717	0.2033	0.4807	0.047*	
C8	0.66304 (11)	0.4819 (2)	0.48013 (10)	0.0343 (4)	
C9	0.60243 (11)	0.6121 (3)	0.50340 (10)	0.0360 (4)	
H9	0.5724	0.5928	0.5495	0.043*	
C10	0.58692 (12)	0.7701 (3)	0.45801 (11)	0.0388 (4)	
H10	0.5463	0.8572	0.4732	0.047*	
C11	0.63252 (12)	0.7973 (3)	0.38967 (10)	0.0366 (4)	
C12	0.69331 (13)	0.6678 (3)	0.36602 (11)	0.0425 (4)	
H12	0.7239	0.6883	0.3203	0.051*	
C13	0.70799 (13)	0.5089 (3)	0.41075 (11)	0.0423 (4)	
H13	0.7475	0.4206	0.3947	0.051*	
C14	0.54518 (11)	0.7736 (3)	0.21080 (10)	0.0373 (4)	
C15	0.51814 (13)	0.5968 (3)	0.20979 (11)	0.0417 (4)	
C16	0.55223 (16)	0.5232 (3)	0.13954 (13)	0.0537 (5)	
C17	0.46521 (19)	0.4975 (3)	0.26793 (16)	0.0662 (7)	
H17A	0.4152	0.4405	0.2394	0.099*	
H17B	0.4453	0.5832	0.3067	0.099*	
H17C	0.5010	0.4045	0.2952	0.099*	
C18	0.5413 (2)	0.3294 (4)	0.10901 (18)	0.0851 (9)	
H18A	0.5263	0.3313	0.0519	0.128*	
H18B	0.4951	0.2697	0.1357	0.128*	
H18C	0.5956	0.2631	0.1198	0.128*	

### Atomic displacement parameters (Å^2^)

**Table:**

	*U*^11^	*U*^22^	*U*^33^	*U*^12^	*U*^13^	*U*^23^
Cl1	0.0529 (3)	0.0449 (3)	0.0932 (5)	0.0139 (2)	−0.0106 (3)	0.0027 (3)
S1	0.0563 (3)	0.0317 (2)	0.0478 (3)	−0.0073 (2)	−0.0044 (2)	0.0078 (2)
O1	0.0551 (8)	0.0479 (8)	0.0507 (8)	0.0055 (7)	0.0206 (7)	0.0053 (6)
O2	0.0592 (9)	0.0676 (11)	0.0756 (11)	−0.0261 (8)	−0.0034 (8)	0.0294 (9)
O3	0.1035 (13)	0.0312 (8)	0.0657 (10)	0.0075 (8)	−0.0100 (9)	−0.0040 (7)
O4	0.0582 (9)	0.0546 (9)	0.0484 (8)	−0.0151 (7)	0.0214 (7)	0.0025 (7)
N1	0.0385 (8)	0.0379 (8)	0.0343 (7)	0.0015 (6)	0.0054 (6)	0.0063 (6)
N2	0.0404 (8)	0.0388 (9)	0.0407 (8)	−0.0005 (7)	0.0033 (7)	0.0074 (7)
N3	0.0833 (14)	0.0628 (13)	0.0581 (11)	−0.0124 (11)	0.0358 (10)	−0.0091 (10)
C1	0.0368 (9)	0.0371 (10)	0.0389 (9)	0.0000 (7)	−0.0002 (7)	0.0046 (7)
C2	0.0434 (10)	0.0396 (10)	0.0352 (9)	−0.0060 (8)	0.0006 (7)	−0.0005 (7)
C3	0.0613 (12)	0.0411 (11)	0.0393 (10)	−0.0094 (9)	0.0056 (9)	0.0045 (8)
C4	0.0646 (13)	0.0362 (10)	0.0456 (11)	−0.0067 (9)	−0.0124 (9)	0.0088 (8)
C5	0.0409 (10)	0.0388 (10)	0.0530 (11)	0.0032 (8)	−0.0103 (8)	0.0002 (9)
C6	0.0382 (9)	0.0440 (11)	0.0495 (11)	0.0044 (8)	0.0050 (8)	0.0056 (9)
C7	0.0380 (9)	0.0399 (10)	0.0402 (9)	0.0019 (8)	0.0071 (7)	0.0053 (8)
C8	0.0344 (8)	0.0353 (9)	0.0329 (8)	−0.0015 (7)	0.0004 (7)	0.0024 (7)
C9	0.0369 (9)	0.0403 (10)	0.0311 (8)	0.0004 (7)	0.0041 (7)	0.0002 (7)
C10	0.0407 (9)	0.0360 (10)	0.0396 (9)	0.0043 (8)	0.0025 (7)	−0.0031 (7)
C11	0.0408 (9)	0.0318 (9)	0.0366 (9)	−0.0027 (7)	−0.0019 (7)	0.0037 (7)
C12	0.0440 (10)	0.0475 (11)	0.0372 (9)	0.0026 (8)	0.0100 (8)	0.0072 (8)
C13	0.0434 (10)	0.0439 (11)	0.0407 (9)	0.0104 (8)	0.0104 (8)	0.0051 (8)
C14	0.0345 (8)	0.0433 (10)	0.0344 (8)	−0.0016 (7)	0.0049 (7)	0.0088 (7)
C15	0.0440 (10)	0.0384 (10)	0.0438 (10)	0.0004 (8)	0.0102 (8)	0.0071 (8)
C16	0.0598 (13)	0.0524 (13)	0.0509 (12)	−0.0057 (10)	0.0174 (10)	−0.0027 (10)
C17	0.0873 (18)	0.0405 (12)	0.0762 (16)	−0.0041 (12)	0.0425 (14)	0.0115 (11)
C18	0.118 (2)	0.0623 (17)	0.0799 (18)	−0.0154 (17)	0.0424 (18)	−0.0195 (14)

### Geometric parameters (Å, °)

**Table:**

Cl1—C5	1.742 (2)	C6—H6	0.9300
S1—O2	1.4238 (17)	C7—H7	0.9300
S1—O3	1.4298 (17)	C8—C9	1.393 (2)
S1—N2	1.6545 (17)	C8—C13	1.395 (2)
S1—C11	1.7630 (18)	C9—C10	1.383 (3)
O1—C2	1.302 (2)	C9—H9	0.9300
O4—C14	1.351 (2)	C10—C11	1.386 (2)
O4—N3	1.419 (2)	C10—H10	0.9300
N1—C7	1.303 (2)	C11—C12	1.393 (3)
N1—C8	1.416 (2)	C12—C13	1.382 (3)
N1—H1	0.87 (2)	C12—H12	0.9300
N2—C14	1.399 (2)	C13—H13	0.9300
N2—H2	0.79 (2)	C14—C15	1.346 (3)
N3—C16	1.305 (3)	C15—C16	1.415 (3)
C1—C6	1.415 (3)	C15—C17	1.487 (3)
C1—C7	1.419 (3)	C16—C18	1.501 (3)
C1—C2	1.431 (2)	C17—H17A	0.9600
C2—C3	1.419 (3)	C17—H17B	0.9600
C3—C4	1.370 (3)	C17—H17C	0.9600
C3—H3	0.9300	C18—H18A	0.9600
C4—C5	1.396 (3)	C18—H18B	0.9600
C4—H4	0.9300	C18—H18C	0.9600
C5—C6	1.361 (3)		
			
O2—S1—O3	121.02 (11)	C13—C8—N1	122.83 (16)
O2—S1—N2	107.38 (10)	C10—C9—C8	120.05 (16)
O3—S1—N2	104.86 (10)	C10—C9—H9	120.0
O2—S1—C11	108.61 (10)	C8—C9—H9	120.0
O3—S1—C11	108.80 (9)	C9—C10—C11	119.31 (17)
N2—S1—C11	105.00 (8)	C9—C10—H10	120.3
C14—O4—N3	106.69 (15)	C11—C10—H10	120.3
C7—N1—C8	126.00 (15)	C10—C11—C12	121.02 (17)
C7—N1—H1	114.5 (14)	C10—C11—S1	119.21 (14)
C8—N1—H1	119.4 (14)	C12—C11—S1	119.64 (13)
C14—N2—S1	119.29 (13)	C13—C12—C11	119.70 (16)
C14—N2—H2	111.7 (17)	C13—C12—H12	120.1
S1—N2—H2	109.3 (16)	C11—C12—H12	120.1
C16—N3—O4	106.39 (16)	C12—C13—C8	119.50 (17)
C6—C1—C7	118.96 (16)	C12—C13—H13	120.3
C6—C1—C2	119.99 (17)	C8—C13—H13	120.3
C7—C1—C2	121.00 (17)	C15—C14—O4	111.35 (17)
O1—C2—C3	121.31 (17)	C15—C14—N2	132.18 (16)
O1—C2—C1	121.30 (17)	O4—C14—N2	116.35 (16)
C3—C2—C1	117.38 (18)	C14—C15—C16	103.91 (17)
C4—C3—C2	120.93 (18)	C14—C15—C17	129.22 (19)
C4—C3—H3	119.5	C16—C15—C17	126.9 (2)
C2—C3—H3	119.5	N3—C16—C15	111.7 (2)
C3—C4—C5	120.87 (18)	N3—C16—C18	121.8 (2)
C3—C4—H4	119.6	C15—C16—C18	126.5 (2)
C5—C4—H4	119.6	C15—C17—H17A	109.5
C6—C5—C4	120.53 (19)	C15—C17—H17B	109.5
C6—C5—Cl1	120.31 (16)	H17A—C17—H17B	109.5
C4—C5—Cl1	119.15 (16)	C15—C17—H17C	109.5
C5—C6—C1	120.25 (18)	H17A—C17—H17C	109.5
C5—C6—H6	119.9	H17B—C17—H17C	109.5
C1—C6—H6	119.9	C16—C18—H18A	109.5
N1—C7—C1	121.81 (16)	C16—C18—H18B	109.5
N1—C7—H7	119.1	H18A—C18—H18B	109.5
C1—C7—H7	119.1	C16—C18—H18C	109.5
C9—C8—C13	120.42 (16)	H18A—C18—H18C	109.5
C9—C8—N1	116.75 (15)	H18B—C18—H18C	109.5
			
O2—S1—N2—C14	56.27 (16)	C9—C10—C11—S1	176.02 (14)
O3—S1—N2—C14	−173.81 (14)	O2—S1—C11—C10	155.29 (15)
C11—S1—N2—C14	−59.20 (15)	O3—S1—C11—C10	21.74 (19)
C14—O4—N3—C16	0.6 (2)	N2—S1—C11—C10	−90.09 (16)
C6—C1—C2—O1	−178.92 (18)	O2—S1—C11—C12	−28.85 (19)
C7—C1—C2—O1	−1.5 (3)	O3—S1—C11—C12	−162.40 (16)
C6—C1—C2—C3	1.4 (3)	N2—S1—C11—C12	85.77 (17)
C7—C1—C2—C3	178.83 (18)	C10—C11—C12—C13	0.6 (3)
O1—C2—C3—C4	177.98 (19)	S1—C11—C12—C13	−175.17 (16)
C1—C2—C3—C4	−2.4 (3)	C11—C12—C13—C8	−1.4 (3)
C2—C3—C4—C5	1.5 (3)	C9—C8—C13—C12	1.3 (3)
C3—C4—C5—C6	0.4 (3)	N1—C8—C13—C12	−179.16 (18)
C3—C4—C5—Cl1	−178.14 (16)	N3—O4—C14—C15	−0.2 (2)
C4—C5—C6—C1	−1.3 (3)	N3—O4—C14—N2	−176.68 (17)
Cl1—C5—C6—C1	177.20 (15)	S1—N2—C14—C15	105.9 (2)
C7—C1—C6—C5	−177.06 (18)	S1—N2—C14—O4	−78.51 (19)
C2—C1—C6—C5	0.4 (3)	O4—C14—C15—C16	−0.2 (2)
C8—N1—C7—C1	−178.19 (17)	N2—C14—C15—C16	175.5 (2)
C6—C1—C7—N1	177.69 (19)	O4—C14—C15—C17	179.5 (2)
C2—C1—C7—N1	0.3 (3)	N2—C14—C15—C17	−4.7 (4)
C7—N1—C8—C9	−174.85 (18)	O4—N3—C16—C15	−0.7 (3)
C7—N1—C8—C13	5.6 (3)	O4—N3—C16—C18	179.6 (3)
C13—C8—C9—C10	−0.5 (3)	C14—C15—C16—N3	0.6 (3)
N1—C8—C9—C10	179.96 (16)	C17—C15—C16—N3	−179.2 (2)
C8—C9—C10—C11	−0.3 (3)	C14—C15—C16—C18	−179.8 (3)
C9—C10—C11—C12	0.2 (3)	C17—C15—C16—C18	0.4 (4)

### Hydrogen-bond geometry (Å, °)

**Table:**

*D*—H···*A*	*D*—H	H···*A*	*D*···*A*	*D*—H···*A*
N1—H1···O1	0.87 (2)	1.85 (2)	2.574 (2)	140 (2)
C7—H7···N3^i^	0.93	2.54	3.429 (3)	161
C12—H12···O2^i^	0.93	2.59	3.391 (3)	145
C17—H17C···O3^ii^	0.96	2.57	3.516 (3)	168
C17—H17B···O1^iii^	0.96	2.38	3.276 (3)	156
N2—H2···O1^iii^	0.79 (2)	2.06 (2)	2.846 (2)	173 (2)

Symmetry codes:  (i) −*x*+3/2, *y*−1/2, −*z*+1/2; (ii) *x*, *y*−1, *z*; (iii) −*x*+1, −*y*+1, −*z*+1.
